# Primary care provider’s barriers to effective management of apparently resistant hypertension in Malaysian public primary health care and strategies to overcome them: a qualitative study

**DOI:** 10.1186/s12875-026-03339-w

**Published:** 2026-04-27

**Authors:** Rafidah Elias, Maila Mustapha, Juslina Omar, Kamarudin Kana, Sabrina Lukas, Imam Bux Brohi, Yu Chun Liu, Chor Yau Ooi

**Affiliations:** 1 Department of Family Medicine, Faculty of Medicine and Health Sciences, Universiti Malaysia Sarawak (UNIMAS), Kuching, Malaysia; 2 Klinik Kesihatan Jalan Masjid, Kuching, Sarawak Malaysia

**Keywords:** Apparently resistant hypertension, Resistant hypertension, Uncontrolled hypertension, Barriers, Challenges, Primary health care

## Abstract

**Background:**

Managing ARH in public PHC is challenging. This study provides the first in-depth qualitative exploration of barriers to effective ARH management among PCPs, including FMS and MO in Malaysian public PHCs, and of strategies to overcome them.

**Methods:**

This qualitative study involved 7 IDIs with FMS and 7 FGDs with small groups of 3 MOs (total 21 MOs), purposively selected across 7 Malaysian public PHCs from March to July 2024. A reflexive thematic analysis grounded in a constructivist paradigm was conducted.

**Results:**

PCPs (FMS and MO) identified barriers at three levels. Theme 1: Patient (Subthemes: poor adherence to medications and follow-up, limited health literacy, inability to afford home BP monitors, culturally driven high-salt diet, lack of family or caregiver support for dependent elderly). Theme 2: Provider (Subthemes: knowledge gaps, diagnostic uncertainty, workload pressures and time constraints, therapeutic hesitancy in complicated cases). Theme 3: Health system (Subthemes: limited diagnostic resources in public PHC, restricted access to FDC antihypertensive medications, vague referral process, limited team-based approach, fragmented care). PCPs (FMS and MO) have employed and proposed strategies to overcome these barriers. Theme 4: Strategies to overcome barriers (Subthemes: engaging family members and caregivers, simplifying out-of-office BP monitoring, optimising clinic appointment scheduling and virtual consultations, establishing multidisciplinary team-based care, professional capacity building, standardising referral algorithms, enhancing patient education materials and programmes, strengthening continuity of care, and improving access to FDC antihypertensive medications).

**Conclusion:**

Addressing these barriers requires healthcare reform centred on multilevel, context-sensitive interventions. Key steps include standardising education and training for patients, caregivers, and PCPs (FMS and MO); standardising referral algorithms; establishing multidisciplinary team-based care; improving access to FDC antihypertensive medications; optimising clinic appointment scheduling and virtual consultations; and strengthening continuity of care.

**Supplementary Information:**

The online version contains supplementary material available at 10.1186/s12875-026-03339-w.

## Background

Uncontrolled hypertension is BP that has not reached target levels despite ongoing management. RH is defined as BP that remains above target despite the concurrent use of three antihypertensive agents from different classes at optimal or maximally tolerated doses, including a diuretic. The definition requires adequate patient adherence and accurate BP measurement [1]. tRH indicates that the elevated BP reflects true biological and pathophysiological resistance to therapy, not artefactual or modifiable causes.

In contrast, ARH refers to patients with uncontrolled BP despite at least three antihypertensive medications. This occurs without confirmed medication adherence, accurate measurement, or exclusion of WCH, and without necessarily verifying optimal pharmacotherapy [2]. These patients appear to have RH based on routine clinical data but may not meet strict diagnostic criteria. Persistently elevated BP in these cases may be due to modifiable factors, such as poor adherence, the white-coat phenomenon, suboptimal dosing, or inaccurate BP measurement, rather than genuine resistance to therapy.

RH and ARH are related but distinct clinical entities. RH denotes treatment resistance confirmed after thorough assessment. ARH reflects uncontrolled BP in clinical practice before tRH is confirmed. Therefore, barriers to managing RH and ARH may differ.

This study aims to examine barriers to effective management of ARH among PCPs (FMS and MO) and strategies to overcome them. The distinction between RH and ARH is important to ensure data validity. The rationale for studying ARH is to capture the practical challenges faced by PCPs (FMS and MO) in everyday practice before resistance is confirmed. In public PHC settings, many patients initially present with ARH before tRH is confirmed.

Additionally, hypertension is highly prevalent in Malaysia. Data from the MyCoSS study indicate that approximately 49% of Malaysian adults have hypertension [3]. Furthermore, the rate of uncontrolled hypertension remains high (44.1%), indicating gaps in effective management [4]. A large proportion of patients in routine practice have ARH. Ineffective management of ARH contributes to uncontrolled hypertension, which is linked to higher cardiovascular risk, target organ damage, and adverse outcomes compared with controlled hypertension [5]. Effective management of ARH can reduce these complications and morbidity.

PHCs in Malaysia form the foundation of the health system and are the first point of contact for most patients seeking healthcare services. PHCs include both the public and private sectors. Although private PHCs outnumber public ones, public PHCs handle a higher patient volume, particularly for chronic disease management. The Ministry of Health Malaysia oversees Malaysia’s public PHCs.

Sarawak, a state in East Malaysia, faces unique contextual challenges that set it apart from other states, particularly those in Peninsular Malaysia. Geographically, clinics are widely dispersed, resulting in long travel times and limited accessibility. Many rural health clinics are in poor physical condition, lacking water, electricity, and internet access, with complex communication and referral processes and limited diagnostic capacity. Sarawak also faces a shortage of doctors and specialists. These differences have important implications for access to care, continuity, and the delivery of chronic disease services, especially for conditions that require consistent follow-up, such as hypertension management.

In Malaysia, public PHC is mainly provided by MO and FMS, who are responsible for managing common chronic illnesses such as hypertension. They are the primary decision-makers for diagnosing and managing hypertension, including ARH. Therefore, understanding barriers to managing ARH requires insights from MO and FMS. Their combined perspectives encompass FMS’s strategic, policy, and leadership insights, as well as MO’s operational, frontline, and experiential insights. While the roles of other healthcare professionals are important in supportive care, they are not primarily responsible for clinical decisions.

There is a shortage of high-quality, dedicated research studies that specifically examine barriers to managing ARH. Many existing studies address medication adherence within ARH, include ARH as part of RH or uncontrolled hypertension investigations, or focus on clinical definitions and management strategies. This points to a research gap on barriers to managing ARH in public PHC. While many barriers to managing ARH overlap with those for RH or uncontrolled hypertension, ARH-specific research remains limited. This highlights the need to explore barriers to managing ARH among PCPs (FMS and MO).

This qualitative study aims to explore barriers to the effective management of ARH among PCPs (FMS and MO) in Malaysian public PHC, as well as strategies to overcome them. The study uses IDI and FGD with PCPs (FMS and MO) to capture in-depth perspectives that quantitative methods cannot fully capture.

## Methods

### Study design

This pivotal qualitative study employed a constructivist paradigm and reflexive thematic analysis to explore the PCP’s barriers to effectively managing ARH and strategies to overcome them. The study involved 7 IDIs with FMS and 7 FGDs with small groups of 3 MOs (for a total of 21 MOs), in line with the developed study protocol.

IDI with FMS offers rich, detailed narratives and captures strategic, policy, and leadership insights, especially in complex decision-making. FGD with MO gathers shared experiences, practical challenges, and feasible strategies through group interaction, reducing overly individualised viewpoints in favour of operational, frontline, and experiential insights of MO. The combination of these two perspectives allows triangulation, enhances credibility, and captures both strategic and operational aspects of managing ARH, providing a comprehensive understanding of barriers and solutions.

### Research team and reflexivity statement

The research team comprises 8 FMSs, including 7 from a public university and 1 from a public PHC. All 7 FMSs from the public university have extensive clinical experience as MOs and FMSs in public PHCs, including managing ARH, before becoming lecturers at the university. The FMS from the public PHC has worked as an MO for 12 years and as an FMS for 15 years.

This shared professional background provided a strong contextual understanding of clinical workflows, therapeutic challenges, health system constraints, and patient-related complexities commonly encountered in public PHC practice. This experiential knowledge enhanced the team’s theoretical sensitivity and facilitated a nuanced interpretation of participants’ accounts. The team actively compared researchers’ perspectives to challenge implicit biases and ensure that findings remained grounded in participants’ narratives rather than in researchers’ preconceptions.

### Study setting and sampling

In Sarawak, PHC is primarily delivered through public health clinics. These clinics employ MOs, staff nurses, assistant MOs, and other health professionals, including pharmacists, dietitians, physiotherapists, and educators. Larger clinics are supervised by an FMS, who manages the teams and handles complex cases. All clinics fall under the jurisdiction of the Sarawak State Health Department (JKNS), which is part of the Ministry of Health Malaysia. In public PHC, most ARH cases are managed by the MO, while FMS handle complex cases.

A purposive sampling method was used to select 7 public PHCs and 30 participants across Sarawak, Malaysia. The public PHCs were selected based on their locations within three zones of Sarawak: the Northern, Middle, and Southern zones, to maximise coverage across the state. Five of the selected public PHCs were in the Southern zone (Kuching and Samarahan), with one in the Middle zone (Sibu) and one in the Northern zone (Miri).

The selected participants comprised 9 FMSs and 21 MOs. They were chosen for their key roles in managing the majority of ARH cases in the public PHC setting. Selection was made by the lead researcher, in collaboration with the FMS/MO in charge of the respective clinic, based on clinical experience in managing ARH and seniority. Priority was given to the most experienced FMS and MO from each clinic to ensure sufficient experience in managing ARH in the public PHC. Other inclusion criteria were at least 1 year of experience managing ARH and the ability to communicate in either English or Malay. Those who did not meet these criteria were excluded. The selected participants were invited via WhatsApp. Interview sessions were scheduled at each participant’s convenience. This applied to both IDI and FGD.

Of the 30 participants approached and accepted (9 FMSs and 21 MOs), only 7 FMSs and all 21 MOs participated in the interview. Two FMSs withdrew from the study: one resigned from the public PHC, and the other was unavailable for an interview during data collection. Recruitment commenced in March 2024 and concluded in July 2024, when data saturation was reached, as determined by team discussion. This indicated that no new codes or themes emerged during the concurrent iterative analysis of both IDI and FGD.

### Data collection

Before the interview session began, all participants received a verbal briefing on the research objectives, an information sheet, and signed an informed consent form. The participants’ information sheet and informed consent form are included as supplementary documents (Additional file 1: Information Sheet & Informed Consent Form). Ethical approvals were obtained from the Medical Research and Ethics Committee (MREC) of the Ministry of Health Malaysia [Reference No: NMRR ID 23-02242-LNJ (IIR)], and from the Director of the Sarawak State Health Department before the site visit. The ethical documents are provided as supplementary files (Additional file 2: MREC Ethical Approval Letter; Additional file 3: Sarawak State Health Department Approval Letter). Participants were informed that they could withdraw from the study at any time and that the interview transcripts would be kept anonymous.

The sociodemographic characteristics of the participants, including age, gender, workplace, years of experience in public PHC, and qualifications, were collected via WhatsApp prior to the interview session.

**Fig. 1:**
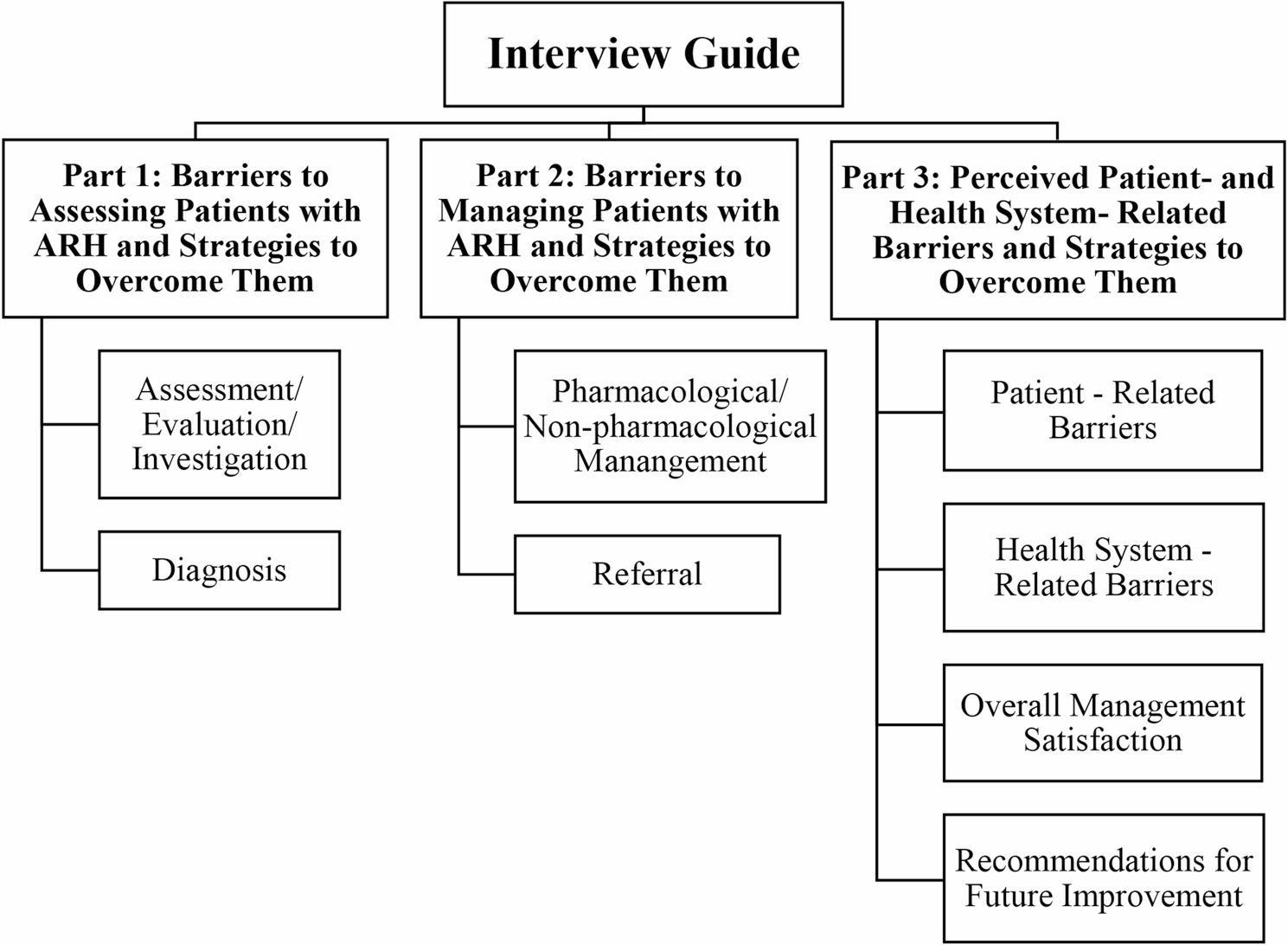
Outline of the interview guide

An English version of a semi-structured interview guide was developed based on the literature and team discussions among all 8 research team members. The same guide was used for both IDI and FGD. The guide was piloted and revised by reorganising and rephrasing questions and by excluding irrelevant ones. The final version is outlined in Fig. 1. The pilot and final versions of the interview guide are provided as supplementary documents (Additional file 4a: Interview guide - pilot; Additional file 4b: Interview guide - final version). Translation into other languages was not carried out because all interviews were conducted in English.

The interview guide comprised three parts (Part 1: PCP’s barriers to assessing patients with ARH and strategies to overcome them; Part 2: PCP’s barriers to managing patients with ARH and strategies to overcome them; Part 3: Perceived patient- and health system-related barriers and strategies to overcome them). Part 1 was subdivided into two sections (assessment/evaluation/investigation and diagnosis). Part 2 was subdivided into two sections (pharmacological/non-pharmacological management and referral). Part 3 was subdivided into four sections (patient-related barriers, health system-related barriers, overall management satisfaction, and recommendations for future improvements). All questions were open-ended.

The interviews were conducted face-to-face in the clinic’s quiet, private consultation room, arranged in advance by the FMS/MO responsible for the respective clinic. Each session comprised one-on-one IDI with an FMS and an FGD with three MOs. All eight members of the research team conducted the interviews. All researchers attended a one-day workshop on conducting qualitative research interviews in March 2024 to prepare for the interviews. Each researcher was assigned to two interview sessions, one IDI and one FGD, as the lead interviewer. Each session was attended by two researchers, one as the lead interviewer and the other as an observer. No other individuals were present during the sessions apart from the interviewers and participants. All interviewers did not have close relationships with the participants to minimise interpersonal conflicts and power dynamics.

The IDI sessions lasted 30–80 min, while the FGD sessions lasted 35–90 min. All sessions were recorded with participants’ permission using an audio recorder. Neither a summary of the interview sessions nor participants’ feedback on the transcript was collected. Data were gathered between March and July 2024 and will be stored in a cloud folder for 5 years before being discarded. No data triangulation was performed.

### Data analysis

Before data analysis began, all audio recordings from interview sessions were transcribed verbatim by professional transcribers. All written transcripts were then checked for accuracy by the respective interviewers. Transcripts from both IDIs and FGDs were anonymised, compiled, and encrypted by the principal investigator (RE) for iterative analysis and review.

Data were analysed using reflexive thematic analysis, as described by Braun and Clarke, within a constructivist paradigm. The analysis was primarily researcher-led, with ChatGPT (OpenAI, GPT-5.3) used as a supplementary tool to support the organisation and structuring of codes and themes.

The research team developed a structured prompt to guide the AI-assisted component of the analysis. The prompt incorporated the study objectives and 8 predefined research questions. It provided explicit instructions to conduct first-cycle descriptive coding across all 7 IDI and 7 FGD transcripts and to apply bracketing principles to ensure that all outputs were strictly grounded in the verbatim transcripts, without introducing assumptions beyond the data.

The 8 research questions were: (1) What are the barriers to conducting the assessment, evaluation, or investigation of ARH among MOs and FMSs in public PHC? (2) What are the barriers to diagnosing ARH among MOs and FMSs in public PHC? (3) What are the barriers to pharmacological management of ARH among MOs and FMSs in public PHC? (4) What are the barriers to non-pharmacological management of ARH among MOs and FMSs in public PHC? (5) What are the barriers to referring ARH among MOs and FMSs in public PHC? (6) What are the perceived patient factors that contribute to ineffective management of ARH among MOs and FMSs in public PHC? (7) What are the perceived health system factors that contribute to ineffective management of ARH among MOs and FMSs in public PHC? (8) What are the strategies to overcome barriers to effective management of ARH among MOs and FMSs in public PHC?

Subsequently, second-cycle coding was conducted to cluster related initial codes into broader categories and to develop higher-order themes aligned with the research questions. Further prompting was used to review, compare and refine themes for consistency, ensuring analytical rigour.

The refined themes and subthemes include Theme 1: Patient-related barriers (Subthemes: poor adherence and medication mismanagement, limited health literacy, socioeconomic constraints, white-coat and pseudo-resistant hypertension, unhealthy lifestyle habits, inadequate family or caregiver support); Theme 2: Provider-related barriers (Subthemes: limited clinical knowledge and confidence, diagnostic inertia, time constraints and workload pressures, fragmented continuity of care, therapeutic hesitancy in complex cases); Theme 3: Health system-related barriers (Subthemes: limited diagnostic resources, restricted access to essential medications, inefficient or unclear referral systems, inadequate access to allied health services, gaps in continuous medical education (CME)); and Theme 4: Strategies to overcome barriers (Subthemes: engaging patients and families, simplified monitoring approaches, scheduling innovations, task sharing and team-based care, professional capacity building).

Moreover, ChatGPT was prompted to provide an excerpt from the transcripts for each theme and subtheme for verification. The provided excerpts were compared with the original transcripts by RE to confirm they were sourced from the transcripts. Excerpts that could not be validated were discarded.

Meanwhile, all eight members of the research team independently reviewed transcripts of their own interviews (one IDI and one FGD), coding line by line and developing themes. Outputs from ChatGPT served only as supplementary references and were critically reviewed by the team. Team members refined, adjusted, or rejected AI-generated suggestions based on their clinical expertise and contextual understanding of public PHC practice. Any disagreements were resolved on the spot during discussion. Final themes, reflecting barriers and strategies, were interpretively constructed by the researchers. This combined method enabled the team to uphold analytical rigour while remaining reflexive about how professional identity, experiential knowledge, and selective AI assistance may have influenced the research process.

To safeguard confidentiality, no identifiable transcripts were uploaded to ChatGPT platforms. All transcripts were anonymised and paraphrased to remove potentially sensitive information about participants and the clinical setting.

### Result

**Fig. 2:**
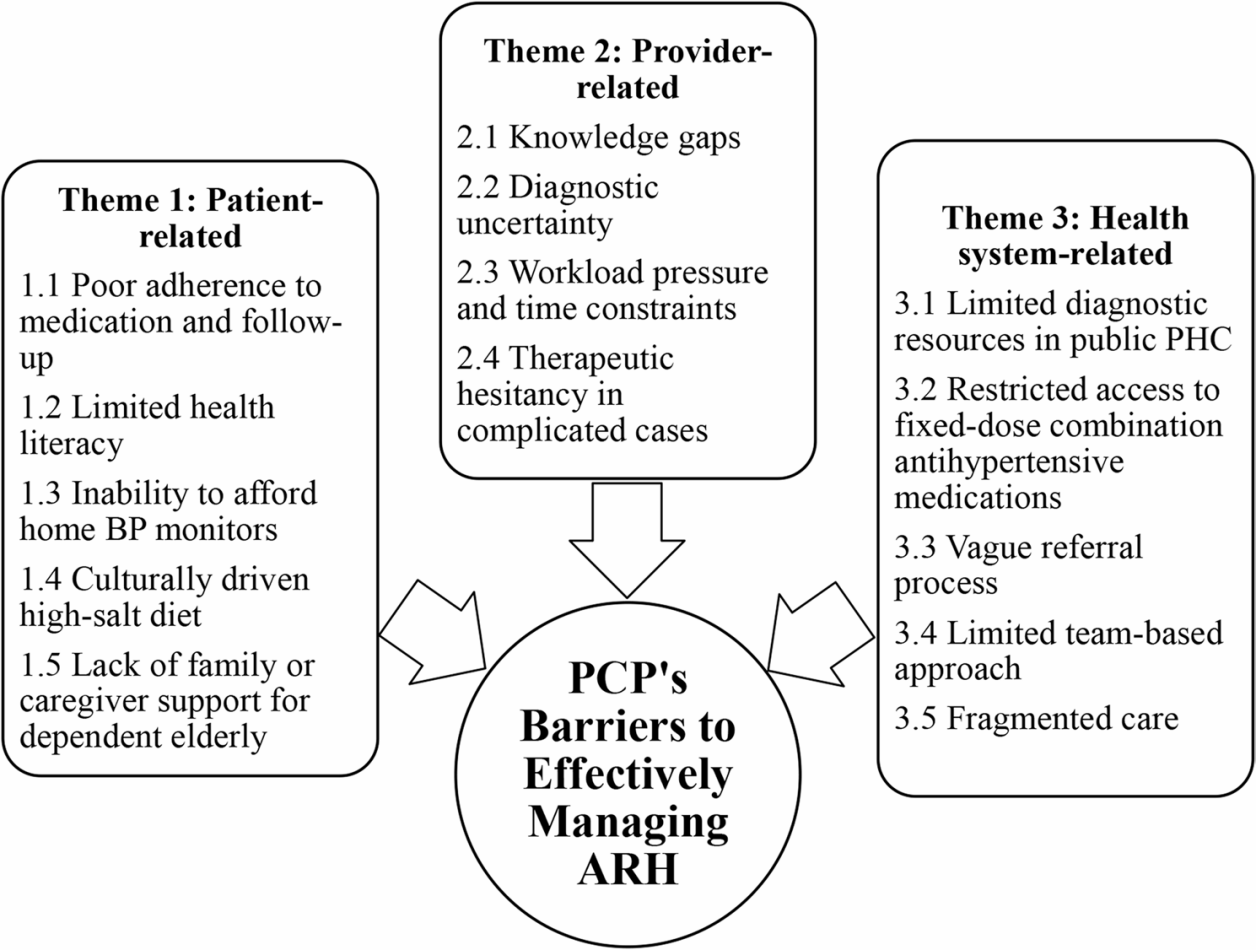
PCP’s barriers to effectively managing ARH within Malaysian public PHC

**Fig. 3:**
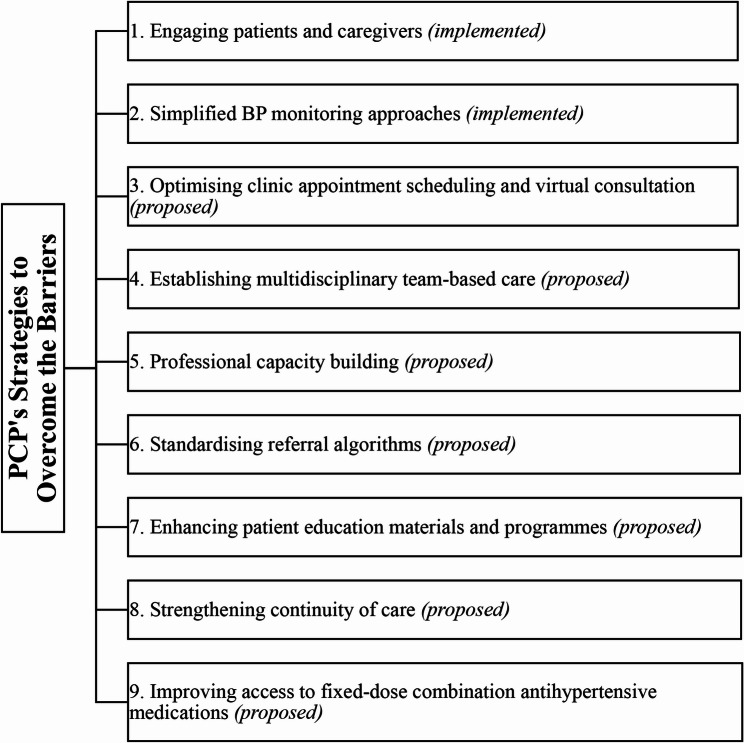
PCP’s strategies (*Implemented/ Proposed*) to overcome the barriers to effectively managing ARH within Malaysian public PHC

A total of 28 participants (7 FMSs and 21 MOs) from 7 public PHCs across Sarawak were enrolled. Most of them (5 FMSs and 15 MOs) came from 5 public PHCs in the southern zone of Sarawak (Kuching and Samarahan). 1 FMS and 3 MOs came from 1 public PHC in the middle zone (Sibu), and another 1 FMS and 3 MOs came from 1 public PHC in the northern zone (Miri), as shown in Tables 1 and 2. The findings are illustrated in Figs. 2 and 3.

**Table 1:** Characteristics of FMSs participating in the IDIs (7 respondents)

*Characteristic*	*n*	%	Mean (SD)
Gender			
Male	2	28.6	
Female	5	71.4	
Age (years)			43.3 (4.61)
Years of practice			12.4 (3.31)

*SD* standard deviation

**Table 2:** Characteristics of the MOs who participated in the FGDs (21 respondents)

Characteristic	*n*	%	Mean (SD)
Gender			
Male	5	23.8	
Female	16	76.2	
Age (years)			35.8 (3.32)
Years of practice			5.8 (2.81)

*SD* standard deviation

### Barriers to effectively managing ARH

#### THEME 1: Patient-related barriers

##### Subtheme 1.1: Poor adherence to medication and follow-up 

Patients often have poor adherence to medications due to forgetfulness, low health literacy, fear of side effects and polypharmacy. They also have poor adherence to follow-up appointments due to work commitments and logistical issues.

*“... there are a lot of medications that they need to remember ...they cannot remember the dosage correctly ...we ask them to take two tablets, but they only take one. Some need to take a twice-daily dose, but they only take a once-daily dose”* *. - FGD6 P24.*

*“... patients say, 'Oh, now the BP has normalised' ...then they disappear and never retake the medication ...thinking that if they continue the medication, they will be forever dependent on the drug”* *- FGD6 P23.*

*“...there are a lot of patients who come and say, 'I haven't been taking this for one year because of certain side effects'...”* *- FGD1 P10.*

*“... it's very difficult for them to come, they have work commitments ...” - FGD2 P13.*

*“...patient defaults again because of the logistic issue ...” - IDI1 P1*.

##### Subtheme 1.2: Limited health literacy

Patients lack awareness of medications and misunderstand their side effects and hypertension, leading to non-adherence to medication, BP self-monitoring and follow-up.

*“…patients cannot identify their medication…” – FGD1 P9.*

*“…some don’t know the real cause of hypertension*,* and they do not understand the indication for starting antihypertensive…when they don’t have the understanding*,* they tend not to adhere to the medication given” - FGD4 P17.*

*“…they don’t want to take so many medications…they think the medication will cause kidney problems” – FGD7 P26.*

*“…the patient’s understanding is…if you give amlodipine*,* she must stop bisoprolol…. -FGD6 P23.*

*“…the awareness is not there*,* and they don’t see the need for a BP machine for the patient who has a BP issue…” - IDI2 P2.*

*“…some of them have poor insight because*,* even though their BP is very high*,* they don’t feel anything…they will wait for symptoms such as dizziness or headache*,* and they will start to worry” - FGD1 P10.*

##### Subtheme 1.3: Inability to afford home BP monitors

Patients often struggle to afford home BP monitors and rely on shared devices and external sources, making it difficult to distinguish WCH from tRH.

*“…they are sharing it with other people…” FGD2 P13.*

*“…difficulty in getting the machine financially…if they have a relative with a BP machine*,* they can just share it” – IDI2 P2.*

*“…most of our patients from lower socioeconomic groups cannot afford a BP machine” - FGD4 P17.*

*“…the BP machine*,* unable to buy*,* unable to do home monitoring” - FGD5 P22.*

*“…if you ask the patient to do home BP monitoring*,* it’s quite difficult because some of them are poor and economic support is not so good…” - FGD6 P23.*

*“…not all the patients have this home BP monitoring…quite difficult for us to assess whether this is a tRH or not” - FGD5 P21.*

##### Subtheme 1.4: Culturally driven high-salt diet

Patients like to eat high-salt local foods.

*“…Iban people usually eat ‘budu’ (fermented anchovies); it’s very salty… but they like to…” – FGD7 P26.*

*“…here we have a lot of salted eggs*,* salted fish*,* and ‘budu’ (fermented anchovies)” – IDI6 P6.*

*“…they like to eat local foods that are high in salt*,* such as salted fish and ‘pekasam’ (fermented fish)” - IDI1 P1.*

*“…elderly people love salted fish and salted eggs” - FGD4 P18.*

*“…in our Asian culture*,* the local Malaysian diet is quite high in salt” - IDI3 P3.*

*“…patients tell me this has been their culture for many years*,* so why change now? If I make my food less salty*,* my husband*,* wife*,* or children will complain” - IDI3 P3.*

##### Subtheme 1.5: Lack of family or caregiver support for dependent elderly

Insufficient support from family or caregivers, especially among dependent elderly individuals, leads to non-adherence, medication errors, and missed follow-ups. Part of this is related to language and communication barriers with older people.

*“…elderly need someone to come and send them for follow-up…” – IDI1 P1.*

*“…elderly live alone…they simply take whatever they remember*,* or none at all…” – FGD6 P24.*

*“…uncle said*,* ‘No one checks on me*,* so I didn’t take my med for 2 months…’” – IDI1 P1.*

*“…elderly*,* when they listen to you*,* do not fully comprehend what’s going on” – FGD3 P16.*

*“…some elderly… we have a language barrier*,* and they didn’t bring their main carer here” – FGD7 P26.*

*“…if patients are too old*,* they don’t have any carers*,* and in terms of how they take their medication*,* we want to ensure they can take it properly*,* which is difficult for me” - IDI7 P6.*

#### Theme 2: Provider-related barriers

##### Subtheme 2.1: Knowledge gaps

PCPs (FMS and MO) have limited knowledge of the causes, investigations, and treatment of ARH in public PHC. They are also uncertain about when to investigate and diagnose secondary hypertension (e.g. phaeochromocytoma, OSA).

*“…SBP is still 160–170 despite 4 medications already; you then wonder which 5th medication to add. This is the difficulty for us” - FGD6 P23.*

*“…if*,* let’s say*,* I suspect the patient has pheochromocytoma… it’s a bit difficult because I’m not really sure how to investigate this… I feel like my knowledge is still not very good” – IDI6 P6.*

*“…we’re not very aware of the signs and symptoms*,* or what kind of condition we will suspect for pheochromocytoma; we are not exposed to it… might not pick it up…” - FGD1 P9.*

*“…my MO… they aren’t aware of ABPM.” - IDI1 P1.*

*“…we don’t know much about how to manage resistant hypertension…” - FGD5 P20.*

*“…we don’t know when to order*,* for example*,* cortisol… I think we need to read more about the secondary causes of hypertension and then try to identify what the possible causes are” - FGD5 P21.*

##### Subtheme 2.2: Diagnostic uncertainty

PCPs (FMS and MO) faced uncertainty in diagnosing RH due to inadequate assessment, missing diagnostic elements from the absence of home BP monitoring/ABPM, and an inability to exclude medication non-adherence.

*“…patients cannot afford a BP machine…difficult to decide whether it is persistent hypertension or WCH…” – FGD4 P17.*

“…when they are not compliant, are they really resistant?” - IDI1 P1.

*“…when I suspected they had office RH, they needed to take BP at home, but they don’t have a BP machine…” – FGD6 P23.*

“…OSA patients, in terms of public PHC, one is limited in time, so we’ll be more reluctant to pursue that diagnosis, including calling the ENT for the sleep study…” - FGD1 P9.

##### Subtheme 2.3: Workload pressure and time constraints

High patient loads and staff shortages impede frequent follow-up, proper assessment and comprehensive patient education and counselling due to time constraints.

*“…too many patients*,* it’s a bit hard to give them the time they need…” – FGD1 P10.*

*“…we have a heavy load of patients and are not able to accommodate frequent follow-ups” - IDI3 P3.*

*“…so many patients*,* and then we don’t have much time to convey the good message to them” - FGD6 P23.*

*“…lack of manpower*,* we have difficulty taking vital signs and correcting BP readings” - FGD6 P24.*

*“…a lot of patients*,* we won’t have much time to further investigate…” - FGD5 P21.*

*“…too many patients*,* they didn’t read through the previous note… missed some investigations and the result is not traced…” – FGD7 P26.*

##### Subtheme 2.4: Therapeutic hesitancy in complicated cases

PCPs (FMS and MO) encounter challenges in optimising medications due to concerns about adherence, side effects and comorbidities such as CKD.

*“…we delay changing medications because we are uncertain whether the treatment has already been fully optimised…” – FGD6 P24.*

*“…if you want to add spironolactone*,* you need to consider the hyperkalaemia risk; advanced CKD is even harder for us…even with the 4th medication*,* BP can remain high…leading you to consider which 5th medication could be added…” – FGD6 P23.*

*“…I will try spironolactone…if they have difficulty coming to monitor their potassium*,* I will consider other medications” – IDI6 P6.*

#### Theme 3: Health system-related barriers

##### Subtheme 3.1: Limited diagnostic resources in public PHC

Public PHC has limited diagnostic resources, primarily ABPM and imaging (e.g., renal Doppler ultrasound), leading to delays in obtaining these tests. Furthermore, hormonal studies often require outsourced testing, which is costly and prone to sample rejection.

*“…24-hour ABPM… the appointment time takes months.” – IDI5 P5.*

*“…ABPM… only PJHUS has the device available.” – IDI1 P1.*

*“…the long waiting time for Doppler is our main issue” - IDI2 P2.*

*“…renal ultrasound*,* very long waiting list*,* 3–4 months” - FGD2 P12.*

*“…24-hour urinary catecholamine*,* we have trouble sending it from public PHC clinics…” - IDI3 P3.*

*“…unable to do investigations in public PHC*,* e.g. 24-hour Dexamethasone suppression test*,* need arrangement” - FGD4 P17.*

*“…blood investigations are sent to an outside lab*,* but it is quite expensive” – IDI1 P1.*

*“…investigations need to be outsourced elsewhere; sometimes your results never come back*,* or they get rejected” – IDI4 P4.*

##### Subtheme 3.2: Restricted access to FDC antihypertensive medications

Restricted access to FDC antihypertensive medications due to quotas, specialist-only prescriptions, irregular supply, and limited stock.

*“…a fixed-dose combination… nowadays we are left with only a few.” – IDI1 P1.*

*“…Coversyl… they will be in stock*,* and at another time there will not be stock.” – FGD1 P9.*

*“…we don’t have many options for a combination pill” – FGD7 P28.*

*“…the stock is very limited*, e.g.,* Coversyl/Coversyl plus… it’s a limited quota” - FGD5 P21.*

*“…we don’t have those triple combinations” - IDI7 P7.*

*“…the quota medication… it’s very hard for us to start the combined medication” – FGD7 P26.*

*“…keep changing the types of medication*,* different generics” - IDI2 P2.*

*“…a single-pill combination is not available for treatment-resistant hypertension” - IDI3 P3.*

##### Subtheme 3.3: Vague referral process

An unclear referral process and unmet expectations among specialists lead to delays and referral rejections.

*“…we’re not sure who we should refer to… medical or FMS… the referral process is not clear-cut” – FGD5 P21*.

*“…Medical were annoyed*,* ‘Why don’t you refer to your FMS first?’” – FGD3 P16.*

*“…Nephrologist says*,* ‘Please ask your FMS to see first… so there’s no clear-cut guideline.’” – FGD3 P14.*

*“…we wonder whether to refer the case or manage it on our side” – FGD6 P23.*

*“…if they say*,* ‘Why don’t you do this first*,* wait for the result*,* and then call us back?’ that will be a problem because the next time you refer*,* it won’t be him*,* and they will ask for something else” - FGD2 P12.*

##### Subtheme 3.4: Limited team-based approach

Healthcare professionals, such as dietitians, pharmacists and counsellors, are under-resourced and not fully integrated into ARH management, limiting team-based care.

*“…dietitians only come once or twice a month…” – FGD7 P27.*

*“…part of the reason we don’t really refer to dietitians is that they are so limited” - FGD1 P9.*

*“…pharmacist sometimes quite busy…dietitian only visits on certain days” - IDI7 P7.*

*“…we don’t have a dietician here…they only come for visits once a month” - FGD6 P24.*

*“…we only have one counsellor to cover the whole clinic” - FGD5 P22.*

##### Subtheme 3.5: Fragmented care

MO rotations disrupt follow-up plans and the doctor-patient relationship.

*“…we rotate MO…at the next visit*,* a different MO might not have noticed the previous plan – FGD1 P9.*

*“…every time the patient comes*,* they see a different MO…medication keeps changing from time to time.” – FGD6 P24.*

*“…we are not seeing the same patient…next time*,* when another doctor sees the patient*,* they didn’t read through my notes…” - FGD7 P26.*

*“…we are rotating MO…we might struggle to build a strong rapport with patients” - FGD1 P8.*

#### Theme 4: Strategies to overcome the barriers

##### Subtheme 4.1: Engaging family members and caregivers

PCPs (FMS and MO) have implemented and proposed engaging with family members and caregivers to gain insights into patients’ medication-taking behaviours and to help elderly, illiterate, and polypharmacy patients understand their medications and maintain adherence.

*“…I will call family members in and ask whether they are truly taking the medication*,* especially the elderly…” – IDI2 P2.*

*“…we can suggest to the carer…to improve their compliance…by arranging their pills in a pill box…” – FGD4 P17.*

*“…the involvement of a family member in reminding them to take the medication or the pill box itself” - FGD1 P8.*

*“…it will be better if we have family members around so that they can supervise the patient in taking the medication” - FGD6 P24.*

*“…elderly patients require a pill box to organise their medication*,* especially if they have more than 5 medications. Patients with dementia need a family member to supervise their medication” - FGD5 P21.*

##### Subtheme 4.2: Simplifying out-of-office BP monitoring

PCPs (FMS and MO) have implemented simplified out-of-office BP monitoring, including photographs of BP readings showing cuff placement and timestamps, as well as BP logs to verify readings. PCPs (FMS and MO) also advised patients to visit a nearby pharmacy or clinic to monitor BP when a home BP monitor is unavailable, to assess WCH/pseudo-RH in resource-limited settings.

*“…take a picture of the BP machine and the cuff*,* with the time of the recording…” – FGD2 P13.*

*“…write a series of BP recordings at home and bring them to me…” – IDI2 P2.*

*“…ask them to drop by any pharmacy or GP that offers FOC BP checking” - FGD2 P13.*

*“…ask them to go to the nearest pharmacy if they don’t have their own BP machine at home. If they have a relative with a BP machine*,* they can share it and record their BP monitoring” - IDI2 P2.*

*“…encourage the patient to go to the nearest clinic to have their BP checked by the MA” – FGD6 P24.*

##### Subtheme 4.3: Optimising clinic appointment scheduling and virtual consultation

PCPs (FMS and MO) proposed optimising clinic appointment scheduling and incorporating virtual consultations to address scheduling inefficiencies.

*“…if sufficient manpower and space*,* we can arrange all appointments within one day…virtual consultations are also an option” – FGD7 P27.*

*“…limit the appointment system…put in a better number of patients per day” - FGD5 P20.*

*“…to have allocated time slots…we are exceeding what we can see” – FGD1 P10.*

##### Subtheme 4.4: Establishing multidisciplinary team-based care

PCPs (FMS and MO) proposed establishing a dedicated team to manage ARH, integrating pharmacists, dietitians, and educators, with standardised protocols to enhance care coordination and minimise ad hoc consultations. This includes collaboration with another speciality, e.g., an endocrinologist.

*“…one-stop centre…create a room…all sorts of medications…file… education…educators…pharmacists…physiotherapists…dietitians are there” – FGD2 P13.*

*“…it’s quite good to establish a team to manage RH. We refer them*,* and they will conduct all investigations and manage the patient accordingly. Once stabilised*,* they will send them back to us” - FGD5 P21.*

*“…a team to just see this type of patient*,* easier for us to refer and for them to receive” - IDI2 P2.*

*“…RH needs a multidisciplinary approach like a team…sitting together*,* seeing a patient” - FGD7 P28.*

*“…collaborate with the endocrine clinic…if they can spare the specialist to come over and manage the patients with us*,* it is a good thing…” – FGD1 P8.*

##### Subtheme 4.5: Professional capacity building

PCPs (FMS and MO) proposed specialist-led CME, state-led guidelines and training to enhance their management skills.

*“…if I can invite an endocrinologist to come and give at least a CME and some guidance on how to manage and investigate*,* I think it will be very helpful so that we have a rough idea of how to investigate from here” - IDI6 P6.*

*“…CMEs are quite important…practising it will be more effective…” – FGD4 P18.*

*“…the state health department can create a guideline for RH…then we go for training…” – FGD1 P9.*

*“…top management should come up with initiatives to improve care…PCPs (FMS and MO) need regular educational activities…” – IDI4 P4.*

##### Subtheme 4.6: Standardising referral algorithm

PCPs (FMS and MO) proposed clear, standardised referral algorithms to enhance communication, streamline referrals, and improve specialist input.

*“…we could reach some consensus with the hospital to develop a referral algorithm” – FGD1 P10.*

*“…if tertiary care implements secular guidelines or an algorithm*,* whenever we refer*,* if it fits the criteria*,* they will accept it instead of having a case-by-case discussion – FGD1 P8.*

*“…if we can sit down with the key person*,* the PCPs (FMS and MO)*,* radiologist*,* nephrologist and cardiologist*,* there must be a flow of referrals from us” - IDI2 P2.*

*“…we need to inform RH of the criteria so they know to refer us earlier and what to do.” – IDI5 P5.*

##### Subtheme 4.7: Enhancing patient education materials and programmes

PCPs (FMS and MO) proposed affordable patient education materials, including videos, pamphlets, and simple digital content, along with an educational programme to engage a wider audience.

*“…we can put a slideshow or whatever on the TV while the patient’s waiting*,* and in the waiting area at the pharmacy*,* give pamphlets to patients.” – FGD3 P15.*

*“…since everyone has handphones*,* they can disseminate information online*,* with a doctor giving simple layman’s teaching about hypertension… educating the public.” – FGD3 P14.*

*“…maybe through the media*,* they can give more information about hypertension… we can provide pamphlets*,* which are useful*,* especially in different languages…” – FGD7 P28.*

*“…more awareness programmes for the community*,* e.g. healthy food exhibition/health promotion corner…” – IDI6 P6.*

##### Subtheme 4.8: Strengthening continuity of care

PCPs (FMS and MO) proposed the family doctor concept and continuity of care to deepen their understanding of patients, build trust, and improve personalised care.

*“…we need a family doctor concept…easier and faster for us to assess the patient’s condition…convinces the patient to take our advice and comply with the medications…” – FGD1 P8.*

*“…their ability to come back for follow-ups helps because I receive more personalised care advice for them…” - IDI3 P3.*

##### Subtheme 4.9: Improving access to FDC antihypertensive medications

PCPs (FMS and MO) proposed improving access to FDC antihypertensive medications to simplify treatment and enhance BP control.

*“…we know that those 3-in-1 single-pill combinations are not easily available*,* and yet we know that making things easier for patients will improve their control…” – IDI3 P3.*

*“…the drug combination is very good for overcoming the pill burden…need to have more choices and get some new drugs…” - IDI6 P6.*

*“…we should have certain medication available in our clinic for a certain group of patients who really need that medication…we do have it on the market*,* but we don’t have it in our public health system*,* which is quite disappointing” - IDI2 P2.*

*“…we can offer more variety*,* especially combinations with three antihypertensives rather than two antihypertensives…” IDI7 P7.*

## Discussion

This study aimed to explore PCPs’ barriers to effective ARH management in Malaysian public PHC, as well as the strategies implemented and proposed to overcome these barriers. The findings revealed a complex interplay of multilevel barriers at the patient, provider, and health system levels, highlighting the complexity of ARH management in Malaysian public PHC.

Under theme 1 (patient-related barriers), five subthemes were identified: poor adherence to medication and follow-up; limited health literacy; inability to afford home BP monitors; culturally driven high-salt diet; and lack of family or caregiver support for dependent elderly.

Under theme 2 (provider-related barriers), four subthemes were identified: knowledge gaps; diagnostic uncertainty; workload pressures and time constraints; and therapeutic hesitancy in complicated cases.

Under theme 3 (health system-related barriers), five subthemes were identified: limited diagnostic resources in public PHC; restricted access to FDC antihypertensive medications; a vague referral process; a limited team-based approach; and fragmented care.

Under theme 4 (strategies to overcome barriers), nine subthemes were identified. Two implemented measures are: engaging family members and caregivers and simplifying out-of-office BP monitoring. Seven proposed measures are: optimising clinic appointment scheduling and virtual consultations; establishing multidisciplinary team-based care; professional capacity building; standardising referral algorithms; enhancing patient education materials and programs; strengthening continuity of care; and improving access to FDC antihypertensive medications.

This study offers new insights that could enhance future management of ARH in Malaysian public PHC. To our knowledge, there is limited evidence that specifically examines the challenges faced by PCPs (FMS and MO) or proposes pragmatic strategies to address barriers to managing ARH within the Malaysian public PHC system.

### Patient-related barriers

Poor adherence to medication and follow-up emerged as a prominent patient-related barrier. This finding aligns with prior studies highlighting medication nonadherence as a leading contributor to uncontrolled hypertension, driven by misconceptions about hypertension and medication side effects [6–8]. Additionally, this study found that forgetfulness, polypharmacy, work commitments, and logistical issues also contributed to non-adherence to medication and follow-up.

Limited health literacy was also a major barrier to adherence, stemming from a lack of understanding of hypertension and medications. This aligns with evidence that low health literacy is associated with poor hypertension outcomes [9–11]. Other studies also report low hypertension awareness and limited availability of effective educational materials in Malaysia [8, 12].

Inability to afford home BP monitors hinders the ability to distinguish tRH from the white-coat effect and pseudo-RH, emphasising the need for broader access to diagnostics for underserved patients [8, 13].

A culturally driven high-salt diet underscores the need for context-specific lifestyle interventions. Chow et al. reported similar findings, with cultural norms influencing salt intake [14].

Additionally, a lack of support from family or caregivers, especially among dependent elderly patients living alone, led to medication errors and non-adherence. Similar concerns have been raised in studies of ageing populations and chronic disease management [15, 16].

### Provider-related barriers

PCPs (FMS and MO) acknowledged knowledge gaps regarding the causes, investigations, and treatment of ARH and secondary hypertension. Limited exposure, insufficient reading on rare causes (e.g., pheochromocytoma), and unawareness of diagnostic resources (e.g., ABPM) have compounded these challenges. This is supported by an earlier study that highlighted the importance of clinician training in diagnosing RH [1].

As noted in this study, diagnostic uncertainty reflects global patterns of hesitancy to escalate therapy [17, 18]. The study highlighted inadequate assessment or missing diagnostic elements, due to the absence of home BP monitoring/ABPM and the inability to exclude medication non-adherence, as a major contributor to diagnostic uncertainty.

Workload pressures and time constraints restrict thorough assessments and in-depth counselling, a problem commonly reported in public PHC systems worldwide [19–21].

Therapeutic hesitancy, particularly in escalating therapy for complex cases, stems from uncertainty about medication adherence, concerns about medication side effects, lack of access to laboratory monitoring, and the presence of comorbidities such as CKD. This mirrors global trends, in which clinical inertia in hypertension care arises from uncertainty, competing risks, and limited time [22].

### Health system-related barriers

A major constraint was limited access to diagnostic resources in public PHC, leading to significant delays in the work-up of suspected secondary causes. Timely diagnostics are fundamental to ARH management [23].

The high cost was reported as a contributor to restricted access to FDC antihypertensive medications, echoing prior findings from Malaysian and regional studies [24].

The referral process is vague, hindering timely specialist input. Referrals often face ambiguity or rejection due to a lack of predefined criteria, resulting in frustration and delayed care. Similar system navigation issues have been documented in other studies [25].

Furthermore, a limited team-based approach, owing to understaffing and a lack of integration, has restricted the implementation of holistic, team-based care models advocated by the WHO [26].

Fragmented care arising from the absence of a family doctor concept compromises rapport and continuity of care, consistent with studies linking continuity of care to better chronic disease outcomes [27].

### Strategies to overcome the barriers

Despite these challenges, the PCPs (FMS and MO) in this study have implemented several strategies and proposed a practical, resource-conscious approach. One strategy was to engage family members and caregivers to improve medication adherence and reduce medication errors. Previous studies have linked this strategy to better overall BP management in resistant cases [28, 29].

The second approach simplified out-of-office BP monitoring by taking photos, keeping BP logs, or visiting pharmacies or nearby clinics. This method uses widely available technologies, such as smartphones and validated home BP monitors, and serves as a practical alternative when ABPM is not feasible [30]. It also provides a larger dataset for interpretation, rather than relying on isolated clinic readings. Numerous studies have shown that out-of-office measurements offer better risk stratification and correlate more closely with organ damage than clinic readings alone [31].

PCPs (FMS and MO) proposed optimising clinic appointment scheduling and incorporating virtual consultations to reduce unnecessary clinic visits while maintaining the quality of care. Evidence from previous studies supports their effectiveness in reducing in-clinic follow-up and wait times, allowing providers to spend more time on complex cases such as ARH and making them comparable to in-person care in hypertension management [30, 32].

Notably, PCPs (FMS and MO) emphasised the importance of multidisciplinary team-based care involving PCPs (FMS and MO), nurses, dietitians, pharmacists, and educators. Previous evidence supports the feasibility and effectiveness of multidisciplinary care in improving BP control, particularly when patients are already struggling with standard care [33, 34].

PCPs (FMS and MO) also emphasised the importance of professional capacity building through specialist-led CME, state-led guidelines, and training to enhance their management skills. Evidence shows that specialist-led CME can enhance PCPs’ capabilities in managing ARH when integrated with supported practice change, case discussions, guideline application, reinforcement, and hands-on tools [35–37]. By contrast, education alone is unlikely to improve patient outcomes.

The suggestion for a standardised referral algorithm is particularly relevant. Recent studies emphasise that structured tools, including referral algorithms, are part of effective strategies to improve the quality and appropriateness of referrals and to enhance patient and staff satisfaction. However, an algorithm alone is unlikely to succeed unless paired with supportive mechanisms such as clinician training on criteria, electronic referral systems or templates, feedback loops between PCPs (FMS and MO) and specialists, and regular audits of referral appropriateness [38].

Furthermore, the PCP’s proposal to develop patient education materials and programmes has been supported by previous studies examining the benefits of digital and multimedia educational resources, traditional materials such as pamphlets, and community-based interventions [39–41]. Moreover, combining these multi-component strategies is more likely to improve hypertension outcomes than any single approach.

PCPs (FMS and MO) advocate the family doctor concept and continuity of care to manage ARH. In theory, the family doctor model might enhance ARH outcomes through holistic, patient-centred care; improved medication adherence through regular medication reviews; seamless information transfer and care planning through continuity of care; and greater opportunities for motivational counselling. However, previous studies have shown mixed results. Despite the theoretical backing, the evidence indicates variability in BP outcomes [27, 42, 43]. This implies that simply ensuring continuity is not sufficient.

Finally, PCPs (FMS and MO) proposed improving access to FDC antihypertensive medications to improve medication adherence, which is a major barrier to ARH management. This has been consistently supported by previous studies [44, 45].

### Strengths and limitations

This study captures perspectives from two clinical roles (FMS and MO) and seven public PHCs across Sarawak on ARH management challenges. Pragmatic strategies proposed by providers were identified.

However, the findings are specific to the public PHC setting in Sarawak. They may be applicable only to similar public PHC settings and not to peninsular Malaysia or private practices. Additionally, patients’ perspectives were not included, which could offer valuable insights for future research.

Further limitations include the initial use of AI for data analysis, which yields preliminary themes and subthemes. However, this was followed by a mitigating measure involving robust human verification of the data and its interpretation.

The future direction and implications for clinical practice involve implementing and refining measures to overcome barriers to managing ARH within public PHC. This includes maintaining current strategies, such as engaging family members and caregivers in ARH care, and simplifying out-of-office BP monitoring as an alternative to ABPM when ABPM is unavailable. These measures must be supported by access to validated BP monitors and patient education. Developing comprehensive, multi-component patient education materials that combine videos, pamphlets, digital platforms, and community initiatives can enhance this support. Clinician education is equally vital and can be strengthened through targeted, structured, long-term, multimodal, specialist-led CME and training. Additionally, establishing well-structured, protocol-based, multidisciplinary team-based care involving PCPs (FMS and MO), nurses, medical assistants, pharmacists, dietitians, and educators is essential. Given the complexity of ARH management, involving specialists in care is critical, and standardised referral pathways should be implemented. Strengthening continuity of care, family doctor models, and optimising clinic appointment scheduling, including virtual consultations, should also form part of the comprehensive approach to managing ARH in public PHC. Finally, FDC antihypertensive medications should be made available in public PHC to improve medication adherence, which remains a major challenge in managing ARH that requires multiple antihypertensive drugs to control BP.

## Conclusion

Managing ARH in public PHCs is hindered by multilevel challenges across the patient, provider, and health system levels. At the patient level, barriers include poor adherence to medication and follow-up, limited health literacy, inability to afford home BP monitors, culturally driven high-salt diets, and lack of family and caregiver support for dependent elderly. At the provider level, barriers include knowledge gaps, diagnostic uncertainty, workload pressures and time constraints, and therapeutic hesitancy in complicated cases. At the health system level, obstacles include limited diagnostic resources in public PHCs, restricted access to FDC antihypertensive medications, a vague referral process, limited team-based approaches, and fragmented care.

Nevertheless, PCPs (FMS and MO) have demonstrated a proactive approach and proposed pragmatic strategies to address these barriers, including engaging family members and caregivers in patients’ care, simplifying out-of-office BP monitoring in the absence of ABPM, optimising clinic appointment scheduling and incorporating virtual consultations, establishing multidisciplinary team-based care comprising PCPs (FMS and MO), nurses, medical assistants, pharmacists, dieticians and educators, building professional capacity through structured, specialist-led CME and training, standardising referral algorithms, enhancing patient education materials and programmes, strengthening continuity of care and improving access to FDC antihypertensive medications.

Addressing these barriers requires healthcare reform centred on multilevel, context-sensitive interventions, including standardising education and training for patients, caregivers, and PCPs (FMS and MO); establishing multidisciplinary, team-based care; standardising referral algorithms; improving access to FDC antihypertensive medications; optimising clinic appointment scheduling and virtual consultations; and strengthening continuity of care. 

## Supplementary Information

Additional file 1. Patient Information Sheet & Informed Consent Form.

Additional file 2. MREC Ethical Approval Letter.

Additional file 3. Sarawak State Health Department Approval Letter.

Additional file 4. Interview Guide – pilot.

Additional file 5. Interview Guide – final version.

## Data Availability

The datasets generated and/or analysed during the current study are not publicly available owing to confidentiality agreements with participants, but are available from the corresponding author upon reasonable request.

## Abbreviations

ABPM: Ambulatory Blood Pressure Monitoring
ARH: Apparently Resistant Hypertension
BP: Blood Pressure
CKD: Chronic Kidney Disease
CME: Continuing Medical Education
FDC: Fixed Dose Combination
FGD: Focus Group Discussion
FMS: Family Medicine Specialist
HBPM: Home Blood Pressure Monitoring
IDI: In-Depth Interview
IMR: Institute for Medical Research
MO: Medical Officer
NCD: Non Communicable Disease
PCP: Primary Care Provider
PHC: Primary Health Care
PJHUS: Sarawak General Hospital Heart Centre
SGH: Sarawak General Hospital
tRH: True Resistant Hypertension
WCH: White Coat Hypertension
