# Supplementary material for: Primary care provider’s barriers to effective management of apparently resistant hypertension in Malaysian public primary health care and strategies to overcome them: a qualitative study
Source: BMC Prim Care. 2026 Apr 27;27:229. doi: 10.1186/s12875-026-03339-w (PMC13255479; doi:10.1186/s12875-026-03339-w)
Supplement: Supplementary file 1 — Additional file 1. Patient Information Sheet & Informed Consent Form. [file 12875_2026_3339_MOESM1_ESM.pdf]

PARTICIPANT INFORMATION SHEET AND INFORMED CONSENT FORM

1. **Title of study:** Barriers to Effective Management of Resistant Hypertension Among Primary Care Doctors in Sarawak: A Qualitative Study

2. **Name of investigator and institution:**

Dr Rafidah binti Elias, Department of Family Medicine, Faculty of Medicine & Health Sciences, UNIMAS

Dr Maila Mustapha, Klinik Kesihatan Jalan Masjid, Kuching, Sarawak

A/P Dr Juslina binti Omar, Department of Family Medicine, Faculty of Medicine & Health Sciences, UNIMAS

Prof. Dr Syed Alwi bin Syed Abdul Rahman, Department of Family Medicine, Faculty of Medicine & Health Sciences, UNIMAS

Prof. Dr Kamarudin bin Kana, Department of Family Medicine, Faculty of Medicine & Health Sciences, UNIMAS

A/P Dr Sabrina Lukas, Department of Family Medicine, Faculty of Medicine & Health Sciences, UNIMAS

A/P Dr Imam Bux Brohi, Department of Family Medicine, Faculty of Medicine & Health Sciences, UNIMAS

A/P Dr Ooi Chor Yau, Department of Family Medicine, Faculty of Medicine & Health Sciences, UNIMAS

A/P Dr Liu Yu Chun, Department of Family Medicine, Faculty of Medicine & Health Sciences, UNIMAS

3. **Name of sponsor:** Medtronic Malaysia Sdn. Bhd.

4. **Introduction**

It is important that you understand why the research is being done and what it will involve. Please take your time to read through and consider this information carefully before you decide if you are willing to participate. Ask the study staff if anything is unclear or if you want more information. After you are properly satisfied that you understand this study and wish to participate, you must sign this informed consent form.

Audio recordings will be taken during the interview. However, during the interview, the audio recording will be de-identified, and there will be no mention of personally identifiable information such as names, IC numbers, etc. The audio recording is for transcription purposes and will not be copied/sent to any other individual or used for any other purpose. After transcription, the audio recording will be disposed of securely.

Your participation in this study is voluntary. You do not have to be in this study if you do not want to. You may also refuse to answer any questions you do not want to answer. You may withdraw from this study at any time. If you withdraw, any data collected from you up to your withdrawal will still be used for the study. Your refusal to participate or withdraw will not affect any medical or health benefits to which you are otherwise entitled.

The Medical Research and Ethics Committee, Ministry of Health Malaysia have approved this study.

5. **What is the purpose of the study?**
   The purpose of this study is to explore the barriers to the effective management of resistant hypertension among primary care doctors in Sarawak. This research is necessary to improve the understanding of barriers to the effective management of resistant hypertension among primary care doctors in Sarawak. This research will be conducted for a duration of 12 months (30/6/23 till 30/6/2024).

6. **What are my responsibilities when taking part in this study?**
   It is important that you answer all the questions asked by the interviewer/moderator honestly and completely, which will take about 30-60 minutes of your time. You will be given a sociodemographic data collection form to be answered. This form will enquire about the name, age, gender, clinic name, and years of working experience as a primary care doctor. Apart from that, you will be interviewed/moderated by one interviewer/moderator among researchers. The interview questions will enquire about your views about barriers to effective management of apparent resistant hypertension.

7. **What are the potential risks and side effects of being in this study?**
   The risk of participation in this study is minimal. You are free to decline to answer any questions you feel uncomfortable with.

8. **What are the benefits of being in this study?**
   The benefit to you is increasing awareness regarding resistant hypertension and barriers to effectively managing the condition. Furthermore, information obtained from this study will help improve the understanding regarding barriers to effective management of resistant hypertension among primary care doctors in primary care centres in Sarawak.

9. **Who is funding the research?**
   This study is receiving outside funding from Medtronic. You will not be paid for participating in this study.

10. **Will my detailed information be kept private?**
    All the detailed information obtained in this study will be kept and handled confidentially, in accordance with applicable laws and/or regulations. Your identity will not be revealed without your consent when publishing or presenting the study results. Individuals involved in this study, qualified monitors and auditors, and governmental or regulatory authorities may inspect the study data, where appropriate and necessary.

11. Who should I call if I have questions?

If you have any questions about the study, please contact the study doctor, Rafidah binti Elias, at telephone number 012-8868485.

If you have any questions about your rights as a participant in this study, please contact The Secretary, Medical Research & Ethics Committee, Ministry of Health Malaysia, at 03-3362 8407/8205/8888.

INFORMED CONSENT FORM

Title of Study: Barriers to Effective Management of Resistant Hypertension Among Primary Care Doctors in Sarawak: A Qualitative Study

By signing below, I confirm the following:

- I have been given oral and written information for the above study and have read and understood the information given.
- I have had sufficient time to consider participation in the study and have had the opportunity to ask questions, and all my questions have been answered satisfactorily.
- I understand that my participation is voluntary, and I can freely withdraw from the study at any time without giving a reason, which will not affect my future treatment. I am not taking part in any other research study at this time. I understand the risks and benefits and freely give my informed consent to participate under the stated conditions. I understand that I must follow the study doctor's (investigator's) instructions related to my participation in the study.
- I understand that study staff, qualified monitors and auditors, the sponsor or its affiliates, and governmental or regulatory authorities have direct access to my record to ensure that the study is conducted correctly and the data are recorded correctly. All personal details will be treated as STRICTLY CONFIDENTIAL
- I will receive a copy of this subject information/informed consent form signed and dated to bring home.

Subject:

Signature:	I/C number:
Name:	Date:

Investigator conducting informed consent:

Signature:	I/C number:
Name:	Date:

Impartial witness:

Signature:	I/C number:
Name:	Date:
