# Supplementary material for: Primary care provider’s barriers to effective management of apparently resistant hypertension in Malaysian public primary health care and strategies to overcome them: a qualitative study
Source: BMC Prim Care. 2026 Apr 27;27:229. doi: 10.1186/s12875-026-03339-w (PMC13255479; doi:10.1186/s12875-026-03339-w)
Supplement: Supplementary file 2 — Additional file 2. MREC Ethical Approval Letter. [file 12875_2026_3339_MOESM2_ESM.pdf]

JAWATANKUASA ETIKA & PENYELIDIKAN PERUBATAN
(*Medical Research & Ethics Committee*)
KEMENTERIAN KESIHATAN MALAYSIA
d/a Kompleks Institut Kesihatan Negara
Blok A, No 1, Jalan Setia Murni U13/52,
Seksyen U13, Bandar Setia Alam,
40170 Shah Alam, Selangor.
Tel: 03-3362 8888/8205

Ref : 23-02242-LNJ
Date: 30-August-2023

RAFIDAH BINTI ELIAS
UNIVERSITI MALAYSIA SARAWAK (UNIMAS)

Dear Sir/ Mdm,

<u>ETHICS INITIAL APPROVAL: NMRR ID-23-02242-LNJ</u>
BARRIERS TO EFFECTIVE MANAGEMENT OF APPARENT RESISTANT HYPERTENSION AMONG PRIMARY CARE DOCTORS IN SARAWAK: A QUALITATIVE STUDY

This letter is made in reference to the above matter.

2. The Medical Research and Ethics Committee (MREC), Ministry of Health Malaysia (MOH) has provided ethical approval for this study. Please take note that all records and data are to be kept strictly **CONFIDENTIAL** and can only be used for the purpose of this study. All precautions are to be taken to maintain data confidentiality. Permission from the District Health Officer / Hospital Administrator / Hospital Director and all relevant heads of departments / units where the study will be carried out must be obtained prior to the study. You are required to follow and comply with their decision and all other relevant regulations.

3. The investigators and study sites involved in this study are:

   <u>KLINIK KESIHATAN TANAH PUTEH</u>
   Rafidah Binti Elias (Penyelidik Utama)

   <u>KLINIK KESIHATAN BANDAR MIRI</u>
   Juslina Omar

   <u>KLINIK KESIHATAN PETRA JAYA</u>
   Syed Alwi Bin Syed Abdul Rahman

   <u>KLINIK KESIHATAN BATU KAWA</u>
   Imam Bux Brohi

   <u>KLINIK KESIHATAN SIBU JAYA</u>
   Sabrina Binti Lukas

   <u>KLINIK KESIHATAN KOTA SENTOSA</u>
   Kamarudin Bin Kana

   <u>KLINIK KESIHATAN KOTA SAMARAHAN</u>
   Ooi Chor Yau

4. The following documents have been received and reviewed with reference to the above study:

1. Study Protocol Version 1, dated 11-July-2023
2. Patient Information Sheet (PIS) & Informed Consent Form (ICF) (English) Version 1.2, dated 8-August-2023
3. Patient Information Sheet (PIS) & Informed Consent Form (ICF) (Malay) Version 1.2, dated 8-August-2023
4. Interview Guideline Version 1, dated 11-July-2023

5. Investigator's documents : Declaration of Conflict of Interest (COI), IA-HOD-IA, and CV:<br>a) Rafidah Binti Elias (Penyelidik Utama)<br>b) Juslina Omar<br>c) Syed Alwi Bin Syed Abdul Rahman<br>d) Imam Bux Brohi<br>e) Sabrina Binti Lukas<br>f) Kamarudin Bin Kana<br>g) Ooi Chor Yau

5. Please note that ethical approval is valid until **29-August-2024**. The following are to be reported upon receiving ethical approval. Required forms can be obtained from the National Medical Research Regisry (NMRR) website:
   i. **Continuing Review Form** has to be submitted to MREC within 2 month (60 days) prior to the expiry of ethical approval.
   ii. **Study Final Report** upon study completion to the MREC.
   iii. Ethical approval is required in the case of **amendments / changes** to the **study documents/ study sites/ study team**. MREC reserves the right to withdraw ethical approval if changes to study documents are not completely declared

6. This study involves the following methods:

   i. Interview

7. Please take note that the reference number for this letter must be stated in all correspondence related to this study to facilitate the process.

Comments (if any): NIL

<u>Project Sites:</u>
KLINIK KESIHATAN TANAH PUTEH
KLINIK KESIHATAN BANDAR MIRI
KLINIK KESIHATAN PETRA JAYA
KLINIK KESIHATAN BATU KAWA
KLINIK KESIHATAN SIBU JAYA
KLINIK KESIHATAN KOTA SENTOSA
KLINIK KESIHATAN KOTA SAMARAHAN

Decision by Medical Research & Ethics Committee:
(√) Approved
( ) Disapproved

Date of Approval : 30-August-2023

DR. NURAIN BINTI MOHD NOOR
Chairperson
Medical Research & Ethics Committee
Ministry of Health Malaysia
MMC No: 31576
