# Supplementary material for: Primary care provider’s barriers to effective management of apparently resistant hypertension in Malaysian public primary health care and strategies to overcome them: a qualitative study
Source: BMC Prim Care. 2026 Apr 27;27:229. doi: 10.1186/s12875-026-03339-w (PMC13255479; doi:10.1186/s12875-026-03339-w)
Supplement: Supplementary file 3 — Additional file 3. Sarawak State Health Department Approval Letter. [file 12875_2026_3339_MOESM3_ESM.pdf]

JABATAN KESIHATAN NEGERI SARAWAK,
JALAN DIPLOMATIK OFF JALAN BAKO,
93050 PETRA JAYA,
KUCHING, SARAWAK,
MALAYSIA.

Telefon : 082-473200
Faks : 082-443053 (Pejabat Pengarah)
: 082-443075 (Pejabat Am.)
: 082-442807 (Pejabat TPKN Kesihatan Awam)

Ruj. Kami : JKNSWK.600-56/12/3 Jld.3(44)
Tarikh : 13 September 2023

Dr. Rafidah binti Elias
Fakulti Perubatan dan Sains Kesihatan
Universiti Malaysia Sarawak

Puan,

MAKLUMBALAS PERMOHONAN KEBENARAN PENGGUNAAN KLINIK KESIHATAN DI SARAWAK UNTUK MENJALANKAN PENYELIDIKAN

Dengan segala hormatnya merujuk kepada perkara di atas. Surat Puan bertarikh 30 Ogos 2023 serta surat daripada Jawatankuasa Etika & Penyelidikan Perubatan bertarikh 30 Ogos 2023 dengan Nombor rujukan 23-02242-LNJ adalah berkaitan.

2. Sukacita dimaklumkan, bahawa pihak Jabatan Kesihatan Negeri (JKN) Sarawak tiada halangan untuk pihak Puan menjalankan Kajian bertajuk ***"Barriers To Effective Management Of Apparent Resistant Hypertension Among Primary Care Doctors In Sarawak: A Qualitative Study"*** di fasiliti seperti berikut:

No.	Nama Fasiliti Kesihatan	Bahagian
1	Klinik Kesihatan Petra Jaya	Kuching
2	Klinik Kesihatan Batu Kawa	Kuching
3	Klinik Kesihatan Tanah Puteh	Kuching
4	Klinik Kesihatan Kota Sentosa	Kuching
5	Klinik Kesihatan Kota Samarahan	Samarahan
6	Klinik Kesihatan Sibu Jaya	Sibu
7	Klinik Kesihatan Bandar Miri	Miri

3. Sehubungan dengan itu, Puan boleh menghubungi Pejabat Kesihatan Bahagian (PKB) yang menjaga Klinik-klinik tersebut untuk menjelaskan berkenaan penyelidikan ini. Mohon kerjasama pihak PKB untuk memantau kajian ini agar perkhidmatan tidak terjejas dan kerahsiaan pesakit dijaga.

4. Seterusnya, hasil kajian tersebut perlu dikongsi dengan JKN Sarawak dan penyelidik perlu memohon kebenaran daripada Kementerian Kesihatan Malaysia sebelum membuat pembentangan awam atau sebarang penerbitan terhadap kajian tersebut.

Perhatian dan kerjasama daripada pihak Puan berhubung dengan perkara di atas amatlah dihargai.

Sekian, terima kasih.

"MALAYSIA MADANI"
"BERKHIDMAT UNTUK NEGARA"

Saya yang menjalankan amanah,

(DR. OOI CHOO HUCK)
MMC NO: 27748
Pengarah
Jabatan Kesihatan Negeri Sarawak

s.k.:

Pegawai Kesihatan Bahagian,
Pejabat Kesihatan Bahagian Kuching.

Pegawai Kesihatan Bahagian,
Pejabat Kesihatan Bahagian Samarahan.

Pegawai Kesihatan Bahagian,
Pejabat Kesihatan Bahagian Sibu.

Pegawai Kesihatan Bahagian,
Pejabat Kesihatan Bahagian Miri.

Site Aprroval Form (B)

MAKLUMBALAS PERMOHONAN KEBENARAN PENGGUNAAN KLINIK KESIHATAN DI SARAWAK UNTUK MENJALANKAN PENYELIDIKAN

Tajuk Penyelidikan : *Barriers To Effective Management Of Apparent Resistant Hypertension Among Primary Care Doctors In Sarawak: A Qualitative Study*

Nama dan Jabatan Ketua Penyelidik : Dr Rafidah binti Elias, Jabatan Perubatan Keluarga, Fakulti Perubatan dan Sains Kesihatan, UNIMAS

Pihak Jabatan Kesihatan Negeri Sarawak dengan ini membuat keputusan seperti berikut : -

- [x] Membenarkan projek penyelidikan dijalankan
- [ ] Tidak membenarkan projek penyelidikan dijalankan

Pihak penyelidik bertanggungjawab dalam memastikan kajian dijalankan secara berintegriti mengikut tatacara yang telah ditetapkan oleh pihak institusi/fasiliti dan Kementerian Kesihatan Malaysia. Pemantauan oleh pihak institusi atau fasiliti/ketua jabatan/CRC unit(sekiranya ada) akan dilakukan dari semasa ke semasa bagi memastikan kajian yang dijalankan adalah berpandukan garis panduan dan pekeliling sedia ada.

Sekian.

Disokong oleh,

(DR. VERONICA LUGAH)
MPM: 36521 NSR: 127845
Pakar Perunding Perubatan Kesihatan Awam
Timbalan Pengarah Kesihatan Negeri
(Kesihatan Awam)
Jabatan Kesihatan Negeri Sarawak

....
(Dr. Veronica Lugah)
Timbalan Pengarah Kesihatan Negeri (Kesihatan Awam)
Tarikh:

Diluluskan oleh,

....
(Dr. Ooi Choo Huck)
Pengarah Kesihatan Negeri Sarawak
Tarikh:

s.k.:
Pegawai Kesihatan Bahagian, Kuching
Pegawai Kesihatan Bahagian, Samarahan
Pegawai Kesihatan Bahagian, Sibu
Pegawai Kesihatan Bahagian, Miri
