# Supplementary material for: Primary care provider’s barriers to effective management of apparently resistant hypertension in Malaysian public primary health care and strategies to overcome them: a qualitative study
Source: BMC Prim Care. 2026 Apr 27;27:229. doi: 10.1186/s12875-026-03339-w (PMC13255479; doi:10.1186/s12875-026-03339-w)
Supplement: Supplementary file 4 — Additional file 4. Interview Guide – pilot. [file 12875_2026_3339_MOESM4_ESM.docx]

**SEMI-STRUCTURED INTERVIEW TOPIC GUIDE**

**TITLE**: BARRIERS TO EFFECTIVE MANAGEMENT OF RESISTANT HYPERTENSION AMONG PRIMARY CARE DOCTORS IN SARAWAK: A QUALITATIVE STUDY

**OBJECTIVES**: To explore barriers to effective management of resistant hypertension among primary care doctors in a primary care centre in Sarawak.

**BEFORE STARTING THE INTERVIEW**

| Component | Example(s) of statement |
| --- | --- |
| 1. Thank you | *Thank you for taking the time to meet with me today.* |
| 1. Introduce | *My name is __, and I would like to talk to you about your experience managing patients with resistant hypertension and the barriers to effectively managing the cases.* |
| 1. Ensure PIS (participant information sheet) is explained |  |
| Purpose/ why the participant has been chosen | *The main aim of this study is to explore barriers to the effective management of patients with resistant hypertension among primary care providers.*  *You have been chosen for this study because we want to explore your experience managing the case and your opinion regarding barriers to effective management.* |
| Expected duration of the interview | *The interview should take less than an hour. I will audiotape the session because I don’t want to miss any of your comments. I can't possibly write fast enough to get it all down. Because we are on tape, please speak up so we don’t miss your comments. Apart from that, I will also take some notes during the session.* |
| Confidentiality | *Even though we are taking notes and audio-taping the session, no real name will be mentioned during the interview, and the interview responses will be kept confidential. That means your interview responses will only be known by the interviewer and data analysing investigators, and we will ensure that any information we include in our report does not identify you as the respondent. Remember you don’t have to talk about anything you don’t want to, and you may end the interview anytime.* |
| 1. Opportunity for questions | *Are there any questions about what I have just explained?* |
| 1. Signature of consent - ask the interviewee to sign the informed consent form | *Are you willing to participate in this interview?* |

**PREAMBLE**

1. ICE-BREAKING
2. BEFORE STARTING THE INTERVIEW, EXPLAIN
   1. There are no right or wrong answers.
   2. It is the experience and opinion that we are interested in
   3. The interview is anonymous; no real name will be mentioned during interview
   4. There is no obligation to answer the questions if they are uncomfortable.
   5. There is a need to audio-record and take notes during the session
   6. Ask if there are questions before starting the interview
3. AT THE BEGINNING OF THE INTERVIEW - REFER THE INTERVIEW QUESTIONS
4. ENDING THE INTERVIEW – REFER TO ENDING/ CLOSING REMARK

**TIPS FOR INTERVIEWERS**

- Build rapport
- Open-ended rather than closed-ended e.g.
  - “Please describe…” instead of “Do you know...”
- Ask factual questions before opinion questions, e.g.,
  - "What were the assessments you did?” then
  - “What assessments did you think were necessary?”
- Use probes as needed. e.g.
  - “Would you explain that further?”
  - “Would you give me an example?”
  - “Is there anything else”
- Prompt if the participant is stuck
  - rephrase the question
  - ask them to recall what they did when facing the situation
  - do not jump in and provide an answer when the respondent is struggling. (you may end up putting words in other people’s mouth)
- Not interrupting the participant
  - wait till the participant has completed the sentence or topic is done if something pops up and you would like to explore (make a note)
- Neutral/ non-judgemental
- One at a time
- Clear words

**INTERVIEW QUESTIONS**

| **PART 1: INTRODUCTION/ BACKGROUND** | | |
| --- | --- | --- |
| **Component** | **Interview Questions** | **Probes** |
| 1. JOB POSITION | Would you tell me your job position? |  |
| 1. WORKING EXPERIENCE | How long have you been working in primary care? |  |
| 1. DEFINITION OF RH | How would you define RH? |  |
| 1. EXPERIENCE IN MANAGING RH | Have you encountered a patient with RH/ persistent hypertension despite three optimal doses of antihypertensive medication, including diuretics? | Would you like to tell me more? |
|  | In your experience, what were the causes of RH that you encountered? |  |

| **PART 2: BARRIERS TO EFFECTIVE MANAGEMENT OF RH** | | |
| --- | --- | --- |
|  | | |
| **PART 2A: KNOWLEDGE/ GUIDELINES** | | |
| **Component** | **Interview Questions** | **Probes** |
| 1. GUIDELINES/   REFERENCES | What guidelines or references have you used to manage RH patients? | If have...  List. Tell me more about it. |
|  |  | If don’t have…  *How about a standard flowchart or treatment protocol for managing patients with RH?*  *Have you ever had specific training or CME regarding managing RH?* |
| 1. CONFIDENCE | How was your confidence in managing RH patients? | Would you tell me more/ explain? |
|  |  |  |
| **PART 2B: EVALUATION/ DIAGNOSTIC INERTIA** | | |
| 1. ASSESSMENT/   EVALUATION | In your experience, what assessments did you do for patients with RH?   1. *History: Ask about compliance, HBPM, anxiety/fear of seeing a doctor/nurse, diet, physical activity, smoking, alcohol, substance use, etc.* 2. *Recheck BP – let the patient rest a while, ensure correct position, cuff size* 3. *Look for secondary causes – obesity, OSA, thyroid disease, phaeochromocytoma, Cushing's syndrome, CKD, primary aldosteronism, renal artery stenosis* | List.  Would you tell me more/ explain it further?  Is there anything else did you do? |
| 1. BARRIERS/ DIFFICULTIES REGARDING ASSESSMENT | What were the barriers/ difficulties that you encountered in performing evaluation/assessment for patients with RH?   1. *Not enough time to explore, high patient burden, no privacy to examine patient, no couch to examine patient, no BP set in the room etc.* | List.  Would you tell me more/ explain it further?  Is there anything else?  Have you overcome the barrier(s)?   - Yes. How did you overcome it? - No. Would you explain why? |
| 1. INVESTIGATIONS | In your experience, have you done any investigation for patients with RH? | Yes.   - List. - Would you tell me more/ explain it further? - Is there anything else?   No   - Would you explain why? |
| 1. BARRIERS/   DIFFICULTIES REGARDING INVESTIGATION | What were the barriers/ difficulties that you encountered in performing investigations for patients with RH?   1. *Familiarity, Availability, Accessibility, Cost* | List.  Would you tell me more/ explain it further?  Is there anything else?  Have you overcome the barrier(s)?   - Yes. How did you overcome it? - No. Would you explain why? |
|  |  |  |
| **PART 2C: MANAGEMENT/ CLINICAL/THERAPEUTIC INERTIA** | | |
| 1. MANAGEMENT | In your experience, how did you manage the patients with RH?   1. *Lifestyle modification/ medications* | List.  Would you tell me more/ explain it further?  Is there anything else? |
| 1. BARRIERS/   DIFFICULTIES REGARDING MANAGEMENT | What were the barriers/ difficulties that you encountered in managing the patient with RH?   1. *Lifestyle modification – patient education, etc.* 2. *Medications - Availability, limited choice, cost, side effects, pill burden* | List.  Would you tell me more/ explain it further?  Is there anything else?  Have you overcome the barrier(s)?   - Yes. How did you overcome it? - No. Would you explain why? |
| 1. REFERRAL | In your experience, have you ever referred the case of RH? | Yes   - Would you tell me more/ explain it further? Why?   No   - Would you explain why? |
| 1. BARRIERS/   DIFFICULTIES REGARDING REFERRAL | What were the barriers/ difficulties that you encountered when referring the patient with RH?   1. *No proper referral processes* 2. *Senior/superior not approachable/ not available* | List.  Would you tell me more/ explain it further?  Is there anything else?  Have you overcome the barrier(s)?   - Yes. How did you overcome it? - No. Would you explain why? |
|  |  |  |
| **PART 2D: SATISFACTION** | | |
| 1. SATISFACTION | In your experience, have you ever had patients with RH that were responsive to your management? | Yes   - To what extent did the patient responsive to the management? - Would you tell me more?   No   - Would you explain why? |
|  | What worked well in your current management of RH? | Would you tell me more? Explain? |
|  | What would you do differently next time? | Would you tell me more? Explain? |
|  |  |  |
| **PART 2E: OTHER BARRIERS AND SUGGESTIONS** | | |
| 1. OTHER BARRIERS/ DIFFICULTIES | *Summarize the barriers that have been mentioned (if you can)*  Have you encountered any other barriers/ difficulties in managing RH besides the one you mentioned? | Yes   - Would you tell me more? - Have you overcome the barrier(s)? - Yes. How did you overcome it? - No. Would you explain why? |
| 1. PATIENT FACTORS BARRIERS/ DIFFICULTIES | In your experience, what were the patient factors contributing to the ineffective management of RH?   1. *patient belief/acceptance,* 2. *medication adherence/side effects,* 3. *lack of social support, low motivation, depression/other cognitive dysfunction, poor coping* 4. *poor lifestyle modification – dietary sodium restriction/exercise/ weight loss, smoking cessation/ alcohol intake reduction/healthy eating plan* 5. *poor patient's education about RH and its management* | List.  Would you tell me more/ explain it further?  Is there anything else?  Have you overcome the barrier(s)?   - Yes. How did you overcome it? - No. Would you explain why? |
| 1. HEALTHCARE SYSTEMS BARRIERS/ DIFFICULTIES | In your experience, what were the healthcare system factors contributing to the ineffective management of RH?   1. *not enough time (high patient burden)* | List.  Would you tell me more/ explain it further?  Is there anything else?  Have you overcome the barrier(s)?   - Yes. How did you overcome it? - No. Would you explain why? |
| 1. RECOMMENDATION/   SUGGESTIONS FOR IMPROVEMENT | What recommendations/suggestions do you have for future improvement in managing RH?   1. *Strategies/ intervention/ tools etc. Tell me more/ justify the reason* | Would you tell me more? Explain? |

| **PART 3: ENDING/ CLOSING REMARK** | | |
| --- | --- | --- |
| ENDING THE INTERVIEW | *We have covered all my questions. Is there anything else you wanted to say* |  |
| REMIND CONFIDENTIALITY |  |  |
| THANK THE PARTICIPANT | *Thank you for your time* |  |
| AUTO-TAPE   - Leave tape running for a few minutes (the “after the interview strip”) |  |  |

**WHAT TO DO FOLLOWING THE INTERVIEW**

1. Check the audiotape for clarity and save it in the research folder
2. Verify information given in interviews as necessary (triangulation of data)
   1. Lab staff
   2. Pharmacist
