# Supplementary material for: Primary care provider’s barriers to effective management of apparently resistant hypertension in Malaysian public primary health care and strategies to overcome them: a qualitative study
Source: BMC Prim Care. 2026 Apr 27;27:229. doi: 10.1186/s12875-026-03339-w (PMC13255479; doi:10.1186/s12875-026-03339-w)
Supplement: Supplementary file 5 — Additional file 5. Interview Guide – final version. [file 12875_2026_3339_MOESM5_ESM.docx]

**SEMI-STRUCTURED INTERVIEW TOPIC GUIDE**

**TITLE**: **BARRIERS TO EFFECTIVE MANAGEMENT OF APPARENT RESISTANT HYPERTENSION AMONG PRIMARY CARE DOCTORS IN SARAWAK: A QUALITATIVE STUDY**

**OBJECTIVES**: To explore the primary care doctors’ experiences, difficulties and challenges in managing patients with apparent resistant hypertension in a primary care centre in Sarawak.

**BEFORE STARTING THE INTERVIEW**

|  | Thank the participant |
| --- | --- |
|  | Introduce yourself (interviewer) & your partner (note-taker) |
|  | Give the PIS (Participant Information Sheet) to the participant   - Read through the PIS with the participant |
|  | Summarise the PIS   - THE MAIN PURPOSE OF THE STUDY: to explore the primary care doctors’ experiences, difficulties, and challenges in managing patients with ARH in the primary care centre in Sarawak - THE REASON WHY YOU HAVE BEEN CHOSEN: you have had experience in managing patients with ARH & we want to explore their experiences, difficulties and challenges in managing patients with ARH - THEREFORE, NO RIGHT OR WRONG ANSWERS: it is your experiences, difficulties and challenges in managing patients with ARH that we are interested to know - THE EXPECTED DURATION OF THE INTERVIEW: should be less than 1 hour - AUDIOTAPING: we will audiotape the session because we do not want to miss   any of your comments   - SPEAK ALOUD: to capture your voice clearly - CONFIDENTIALITY: the audio recording will be de-identified; that means no real name will be mentioned in the transcription. The audio recording will be disposed of after transcription. The transcription will be kept confidential and only accessible to the data analysing investigators. We will ensure that any information we include in our report will not identify you as the respondents |
|  | Any questions? |
|  | Sign the Informed Consent Form (2 copies)   - 1 for participant & 1 for researcher |

**INTERVIEW QUESTIONS**

***Start the audiotaping***

***Start with the recording by saying, “This is the IDI (in-depth interview)/FGD (focus group discussion) session (1 or 2) at KK—"***

| **INTRODUCTION** | |
| --- | --- |
| **Component** | **Interview Questions** |
| 1. **JOB POSITION** | Tell me about your job position. |
| 1. **WORKING EXPERIENCE** | How long have you been working in primary care? |
| 1. **EXPERIENCE IN MANAGING RH** | Have you personally managed patients with ARH or patients with persistent hypertension despite 3 optimal doses of antihypertensive medications, including diuretics? |

Thank you for the information.

The next interview questions focus on your **EXPERIENCES, DIFFICULTIES AND CHALLENGES IN ASSESSING AND MANAGING** patients with ARH. It is divided into THREE PARTS

1. Your experiences, difficulties and challenges in assessing patients with ARH
2. Your experiences, difficulties and challenges in managing patients with ARH
3. Patients and healthcare systems barriers in managing patients with ARH, your satisfaction and recommendation

Remember. There are **NO RIGHT OR WRONG ANSWERS**.

We are interested in **YOUR EXPERIENCES, DIFFICULTIES, AND CHALLENGES** in managing patients with ARH.

Now we start with PART 1 of the interview questions regarding **YOUR EXPERIENCES, DIFFICULTIES AND CHALLENGES IN ASSESSING** patients with ARH.

| **PART 1: ASSESSMENT/ EVALUATION/ INVESTIGATION/ DIAGNOSTIC INERTIA**  In this part 1, there are 2 components that I will ask. The first component is regarding assessment/evaluation and investigation. The second component is regarding diagnosis. | | |
| --- | --- | --- |
| **Component** | **Main Interview Questions** | **Probing Questions** |
| 1. **ASSESSMENT/ EVALUATION/ INVESTIGATION** | 1. Based on your experience in managing patients with ARH, what assessment/evaluation/investigation did you do for patients with ARH or persistent hypertension despite 3 optimal doses of antihypertensive medications, including diuretics? | Is there anything else? |
|  | 1. How did you do it? |  |
|  | 1. *List down the assessment*.   You said that you did …  What were the difficulties or challenges that you encountered in doing the assessment/evaluation/investigation you just mentioned? | **IF NO**  Why didn’t you encounter any difficulties or challenges? |
|  | 1. How did you overcome the difficulties or challenges that you encountered? | **IF NO**  Why didn’t you overcome it?  In your opinion, what can be done to overcome it? |
| 1. **DIAGNOSIS** | 1. Based on your experience, what were the underlying causes of ARH that you encountered? |  |
|  | 1. How did you diagnose/ find out the underlying causes? |  |
|  | 1. *List down the underlying cause of ARH.*   You said the underlying causes were… and you diagnosed it based on …  What were the difficulties or challenges that you encountered in diagnosing/finding the underlying cause of ARH you just mentioned? | **IF NO.**  Why didn’t you encounter any difficulties/ challenges? |
|  | 1. How did you overcome the difficulties or challenges that you encountered? | **IF NO**.  Why didn’t you overcome it?  In your opinion, what can be done to overcome it? |

Thank you for your responses.

We have completed the Part 1 of the interview questions.

LOOK @ PARTICIPANT: Is there anything else you wanted to add?

LOOK @ NOTE-TAKER: Is there any questions you wanted to ask?

Now we move on to Part 2 of the interview questions regarding **YOUR EXPERIENCES, DIFFICULTIES AND CHALLENGES IN MANAGING patients with ARH.**

| **PART 2: MANAGEMENT/ CLINICAL/ THERAPEUTIC INERTIA**  In this part 2, there are 2 components that I will ask. The first component is regarding management, and the second component is regarding referral. | | |
| --- | --- | --- |
| **Component** | **Main Interview Questions** | **Probing Questions** |
| 1. **MANAGEMENT**   **(non-pharmacotherapy/ pharmacotherapy)** | 1. Based on your experience, how did you manage patients with ARH that you encountered? | Is there anything else? |
|  | 1. *List down the management*   You said that you did …  What were the difficulties/challenges you encountered while managing the case? | **IF NO.**  Why didn’t you encounter any difficulties/ challenges? |
|  | 1. How did you overcome the difficulties/challenges you encountered while managing the case? | **IF NO.**  Why didn’t you overcome it?  In your opinion, what can be done to overcome it? |
| 1. **REFERRAL** | 1. Based on your experience, to whom did you refer the patient with ARH that you encountered? | **IF NO.**  Why didn’t you refer the case?  When would you refer the case? |
|  | 1. How did you make the referral? |  |
|  | 1. *List the referral process.*   You said that you referred …  What were the difficulties/ challenges you encountered while making the referral? | **IF NO**.  Why didn’t you encounter any difficulties/ challenges while making the referral? |
|  | 1. How did you overcome the difficulties/ challenges you encountered while making the referral? | **IF NO**.  Why didn’t you overcome it?  In your opinion, what can be done to overcome it? |

Thank you for your responses.

We have completed the Part 2 of the interview questions.

LOOK @ PARTICIPANT: Is there anything else you wanted to add?

LOOK @ NOTE-TAKER: Is there any questions you wanted to ask?

We will move on to Part 3, the last part of the interview questions regarding **PATIENTS AND HEALTHCARE SYSTEM BARRIERS TO EFFECTIVE MANAGEMENT OF ARH, YOUR SATISFACTION AND RECOMMENDATION**.

| **PART 3: PATIENTS & HEALTHCARE SYSTEM BARRIERS, SATISFACTION & RECOMMENDATION**  In part 3, there are 4 components that I will ask. The first component is regarding the patient barrier; the second component is regarding the healthcare system barrier, the third component is regarding your satisfaction, and the last component is regarding your recommendation. | |  |
| --- | --- | --- |
| **Component** | **Main Interview Questions** |  |
| 1. **PATIENTS** | 1. Based on your experience, what were the patient factors contributing to the ineffective management of ARH? |  |
|  | 1. *List the patient’s factors.*   You said that patient’s factors were …  How did you overcome the patient factors you just mentioned | **IF NO.**  Why didn’t you overcome it?  In your opinion, what can be done to overcome it? |
| 1. **HEALTHCARE SYSTEM** | 1. Based on your experience, what were the healthcare system factors contributing to the ineffective management of ARH? |  |
|  | 1. *List the healthcare system factors*.   You said that healthcare system factors were …  How did you overcome the healthcare system factors you just mentioned | **IF NO.**  Why didn’t you overcome it?  In your opinion, what can be done to overcome it? |
| 1. **SATISFACTION** | 1. Were you satisfied with the current management of patients with ARH, and why? |  |
| 1. **RECOMMENDATION** | 1. What is your recommendation for future improvement in managing patients with ARH? Explain your recommendation |  |

**ENDING/ CLOSING REMARK**

|  | **ENDING THE INTERVIEW**   - Thank you very much for your responses - We have completed all the interview questions - Is there anything else you wanted to say |
| --- | --- |
|  | **REMIND CONFIDENTIALITY**   - The interview responses will be kept confidential - It can only be accessed by the data analysing investigators - We will ensure that any information we include in our report does not identify you as the respondent. |
|  | **THANK THE PARTICIPANT** |
|  | **AUDIOTAPE**   - *Leave it running for a few minutes after the end of the interview before stopping the recording/ “after the interview strip.”* - *Check the audiotape for clarity and save it in the research folder* |
